# Highly efficient modulation doping: A path toward superior organic thermoelectric devices

**DOI:** 10.1126/sciadv.abl9264

**Published:** 2022-03-30

**Authors:** Shu-Jen Wang, Michel Panhans, Ilia Lashkov, Hans Kleemann, Federico Caglieris, David Becker-Koch, Jörn Vahland, Erjuan Guo, Shiyu Huang, Yulia Krupskaya, Yana Vaynzof, Bernd Büchner, Frank Ortmann, Karl Leo

**Affiliations:** 1 Dresden Integrated Center for Applied Physics and Photonic Materials (IAPP), Technische Universität Dresden, 01069 Dresden, Germany.; 2 Leibnitz Institute for Solid State and Materials Research, IFW, Helmholtzstraße 20, 01069 Dresden, Germany.; 3 Technische Universität München, Department of Chemistry, Lichtenbergstr. 4, 85748 Garching b. München, Germany.; 4 Center for Advancing Electronics Dresden (cfaed), Technische Universität Dresden, Helmholtz Str. 18, 01069 Dresden, Germany.; 5 CNR-SPIN Institute, Corso F. M. Perrone 24, 16152 Genova, Italy.

## Abstract

We investigate the charge and thermoelectric transport in modulation-doped large-area rubrene thin-film crystals with different crystal phases. We show that modulation doping allows achieving superior doping efficiencies even for high doping densities, when conventional bulk doping runs into the reserve regime. Modulation-doped orthorhombic rubrene achieves much improved thermoelectric power factors, exceeding 20 μW m^−1^ K^−2^ at 80°C. Theoretical studies give insight into the energy landscape of the heterostructures and its influence on qualitative trends of the Seebeck coefficient. Our results show that modulation doping together with high-mobility crystalline organic semiconductor films is a previosly unexplored strategy for achieving high-performance organic thermoelectrics.

## Teaser

Highly efficient modulation doping is realized in ordered organic semiconductors enabling high performance thermoelectric devices.

## INTRODUCTION

Efficient doping for charge-carrier creation is key in semiconductor technology. For silicon, efficient doping by shallow impurities was already demonstrated in 1949 (*1*). In the development of further semiconductor technologies, efficient doping has been the most important, e.g., for the GaN technology that enabled the light-emitting diode lighting revolution (*2*, *3*). Recently, carbon-based organic semiconductors have drawn much attention due to their attractive application perspectives for flexible, lightweight, and environmentally friendly electronics (*4*). The most successful product so far is the organic light-emitting diode display with a multibillion U.S. dollar market, which are using doping by controlled coevaporation of small-molecule semiconductors and dopant molecules (*5*). The microscopy nature of doping in organic semiconductors is strongly different from inorganic semiconductors (*6*). One particularly relevant difference is that the dopant concentrations in organic are usually orders of magnitude higher than in inorganics to saturate the high level of deep traps in these materials (*7*). Consequently, one often ends in the so-called reserve regime, where doping efficiency is low (*8*).

Modulation doping is a widely used doping method in inorganic semiconductors where a heavily doped wide bandgap semiconductor is brought in contact with a narrow bandgap semiconductor. Efficient doping at the heterostructure interface is achieved by charge transfer from the wide bandgap semiconductor to the narrow bandgap semiconductor. The main advantage of this doping technique is the avoidance of ionized impurity scattering in the undoped narrow bandgap semiconductor. Hence, both carrier concentration and mobility can be independently maximized (*9*–*11*). Despite the wide use of modulation doping in high-frequency inorganic electronics, this doping technique has not been well studied and exploited in organic semiconductors (*12*, *13*). In particular, it has not been proven that higher doping efficiencies can be achieved. Furthermore, the proof-of-principle studies have not yet demonstrated improvements in devices or at least device-relevant parameters (*12*, *13*).

One area where these improvements are needed is thermoelectrics. Thermoelectric materials have been extensively investigated over the past few decades due to the wide applicability of thermoelectric generators and the ease of energy harvesting from heat sources (*14*, *15*). The performance of the thermoelectric material is distinguished by a figure of merit

$$\mathrm{ZT}=S^{2}\sigma T/k$$ (1)

where *S*, σ, *T*, and *k* correspond to Seebeck coefficient, electrical conductivity, temperature, and thermal conductivity, respectively (*14*, *15*). The nontrivial relationship between the parameters that govern the figure of merit often results in a trade-off between them for optimal thermoelectric performance.

Organic materials could excel in thermoelectrics owing to their potential for low cost and large-area fabrication as well as their low thermal conductivity (*16*, *17*). They could provide a solution for powering small, autonomous devices such as flexible tags or could help to increase the energy efficiency of handheld devices, e.g., by harvesting the heat released from a mobile phone. Given their remarkably low thermal conductivity values, the thermoelectric power factor (*S*^2^σ) is the key figure of merit for optimizing organic thermoelectrics.

The electrical conductivity σ can be tuned by several orders of magnitude by doping; however, this also lowers the Seebeck coefficient as the chemical potential of the host-dopant system is reduced with increasing dopant concentration. Consequently, electrical conductivity and Seebeck coefficient need to be balanced to maximize the figure of merit *ZT*. The interplay between doping, charge carrier transport, and disorder in organic semiconductors, however, is a complex matter since the charge carrier mobility, the Seebeck coefficient, and the doping efficiency are connected to the degree of order/disorder in the system. In particular, at high dopant concentrations [beyond 10 mole percent (mol %)], doping influences the structural order of the host material, reducing the charge carrier mobility and consequently conductivity (*18*–*20*). On the other hand, the reduction of structural order and hence an increased width of the density of states of the host might increase the doping efficiency (*6*). For very high dopant concentrations though, the increased electrostatic disorder effectively reduces the doping efficiency and limits the number of free charge carriers in the doped layer (*6*, *21*). Obviously, modulation doping of organic materials, particularly materials with high mobilities, is a very promising method to find new ways to optimize thermoelectric parameters.

In this work, we show that vacuum-processed high-mobility rubrene thin-film crystals can be efficiently modulation-doped. We prove that the high doping efficiencies can be realized even for high dopant concentrations, avoiding the reserve regime for conventional doping. We study the thermoelectric properties of different polymorphs of rubrene thin-film crystals with modulation doping. We observe a substantial improvement of the thermoelectric parameters. The thermoelectric characteristics are rationalized with comprehensive simulations.

## RESULTS AND DISCUSSION

### Modulation doping efficiency

**Fig. 1.**
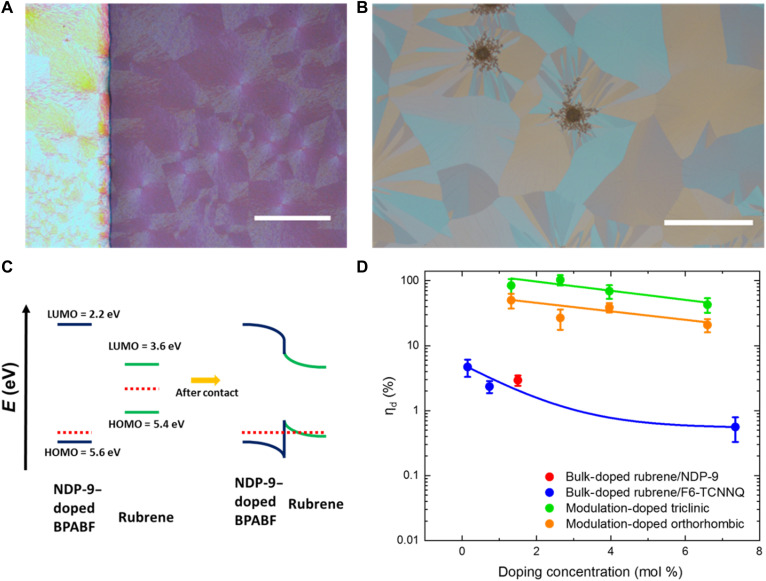
Modulation doping mechanism and doping efficiency. (**A**) Polarized microscope images of a triclinic crystalline rubrene thin film (50 nm thickness) and (**B**) a orthorhombic rubrene platelet thin film (40 nm)/5-nm TAPC underlayer grown on glass substrates. Scale bars, 500 um. (**C**) Energy level diagram of NDP-9–doped 9,9-*bis*[4-(*N*,*N*-*bis*-biphenyl-4-yl-amino)phenyl]-9H-fluorene (BPAPF) and rubrene and the interface between NDP-9–doped BPAPF and rubrene. The red dotted line denotes the chemical potential. Energy values are given with respect to the vacuum level. LUMO, lowest unoccupied molecular orbital. (**D**) Doping efficiency (ionized dopant, η_d_ = *N*_A_^−^/*N*_A_) as a function of dopant molar concentration determined from Mott-Schottky analysis. The green and orange circles denote modulation-doped triclinic and orthorhombic rubrene thin-film crystals with BPAPF:NDP-9, respectively. The red circle represents bulk-doped orthorhombic rubrene thin-film crystals with NDP-9. The bulk-doped triclinic rubrene/F6-TCNNQ data (blue circles) were taken from (*21*) for comparison. The blue and orange solid lines are guides for the eyes.

Rubrene is a well-known high mobility organic semiconductor with carrier mobility exceeding 10 cm^2^ V^−1^ s^−1^ (*22*, *23*) and is consequently an interesting material system for thermoelectrics. Furthermore, it has a number of polymorphs and can be transformed from amorphous thin films into large-area crystalline thin films by a simple thermal annealing method, which allows us to study the charge and thermoelectric transport in a single material system with tunable electronic properties (*24*–*27*). In this work, we grow large-area polycrystalline triclinic (Fig. 1A) and orthorhombic platelet (Fig. 1B) rubrene thin-film crystals on glass substrates by adjusting the annealing temperature, time, and underlayer material (see Materials and Methods). The detailed microstructural characterizations of these crystals have been carried out in our previous work (*26*, *27*). The ideal herringbone molecular packing of the orthorhombic rubrene crystal results in strong electronic overlap between frontier orbitals and hence excellent charge transport in all spatial directions (*28*). On the other hand, the triclinic rubrene crystals show highly anisotropic charge transport with excellent vertical and poor lateral transport due to the strongly branched nature of the crystals (*27*).

As we have previously shown, these rubrene crystals can be p- or n-type doped on demand by coevaporation, allowing us to increase the electrical conductivity up to values of 0.1 S/cm (*26*, *27*). Despite the possibility of directly doping the rubrene crystals, this approach is not favorable for thermoelectric devices as the doping efficiency is low in the crystalline host (which will be discussed later), and doping restricts the size of crystallites formed during the annealing process (*26*). As an alternative to direct doping, we achieve modulation doping in rubrene thin-film crystals by interfacing them with a thin layer of the hole transport material, 9,9-*bis*[4-(*N,N*-*bis*-biphenyl-4-yl-amino)phenyl]-9H-fluorene (BPAPF) doped with the strong p-type dopant NDP-9. The deep highest occupied molecular orbital (HOMO; 5.6 eV) of BPAPF allows the transfer of free holes into the rubrene HOMO level (5.4 eV), resulting in free charge carriers at the chemical potential as shown in Fig. 1C. We have chosen BPAPF as the wide energy gap host material due to the possibility to achieve doping efficiency close to unity in these amorphous hole transport materials (*6*, *13*). To quantify the charge transfer rate and the doping efficiency, we use impedance spectroscopy that is a commonly used method in this regard (*13*, *29*). The charge depletion width at an ideal unipolar junction can be modulated by an external applied voltage that results in a changing capacitance with a linear 1/*C*^2^ versus *V* relationship. The density of ionized dopant, *N*_A_^−^, can then be extracted from the Mott-Schottky relation (Eq. 2)

$${N_{A}}^{-}=\frac{2}{qA^{2}\varepsilon_{0}\varepsilon_{r}m}$$ (2)

where *q* and *A* denote the elementary charge and the device area, respectively. ε_0_, ε*_r_*, and *m* are the vacuum permittivity, the relative permittivity (for BPAPF ~3.5), and the slope of 1/*C*^2^ versus *V*, respectively.

We have built Schottky and hetero-junction diodes using the modulation doping architecture to characterize the density of ionized dopants present in the hetero-junction (the stack geometry and typical *CV* measurements are shown in fig. S1). The charge transfer efficiency from the ionized dopants to rubrene is estimated to be 100% due to the energy level offset and the large gradient of charges (*13*) as verified by our conductivity and field-effect transistor measurement (fig. S2). Figure 1D shows the doping efficiency (estimated density of free hole carriers in rubrene crystals divided by total density of dopants) as a function of the dopant concentration (density of ionized dopant shown in fig. S3). The doping of rubrene crystals by modulation doping is highly efficient as indicated by the doping efficiency of >20% and the fact that the doping efficiency does not show a major reduction with increasing dopant concentration. As a comparison, the doping efficiency of bulk-doped triclinic rubrene crystals is also shown in Fig. 1D (*27*). It can be seen that the doping efficiency of the bulk-doped orthorhombic and triclinic rubrene crystals is not only far lower than for modulation-doped rubrene crystals but also drops rapidly with increasing dopant concentration, which we ascribe to entering the reserve regime (*8*). For highly ordered rubrene crystals, only limited amount of dopants can be introduced into the crystal lattice without deteriorating its structure, hence leading to an increase in the electrostatic disorder with limited charge-transfer states interactions. The increasing electrostatic disorder and minimal charge-transfer states interaction limits successful charge transfer from the dopant to the host molecule in the rubrene crystal lattice as dopants are introduced (*6*–*8*). Although organic crystals are typically associated with narrow density of states making efficient doping challenging, the modulation doping approach shows excellent doping efficiency due to an energetic offset that favors charge transfer from doped BPAPF to rubrene and minimal electrostatic disorder in rubrene.

### Charge and thermoelectric transport

**Fig. 2.**
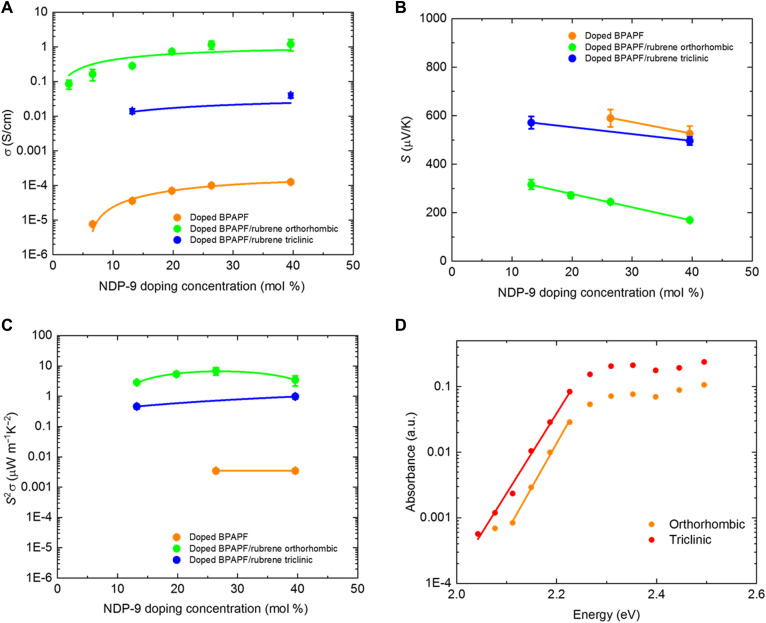
Thermoelectric and photothermal deflectivity measurements of modulation-doped rubrene crystalline thin films. (**A** to **C**) Conductivity, Seebeck coefficient, and thermoelectric power factor dependence on the doping concentration. The conductivity and Seebeck coefficients were taken at 30°C. The blue, green, and orange solid lines are guides for the eye. (**D**) Photothermal deflection spectroscopy of triclinic (*U*_e_ = 82 ± 4 meV) and orthorhombic platelet (*U*_e_ = 74 ± 3 meV) rubrene crystalline thin films. The straight lines are fits of the Urbach tails. a.u., arbitrary units.

The lateral conductivity of both the modulation-doped rubrene and bulk-doped BPAPF showed a rapid increase at low doping (around 5 mol %) followed by steady subsequent increase with further increase of the dopant concentration (Fig. 2A). This trend represents the transition from the trap-filling regime at low dopant concentration to a trap-free regime at higher dopant concentration in BPAPF [also observed in other material systems (*29*)]. In addition, the conductivity of the modulation-doped rubrene crystals is orders of magnitude higher than for the bulk-doped BPAPF layers. This proves successful transfer of free charge carriers into the rubrene crystals; the doped BPAPF layers have a negligible contribution to the charge transport in the heterostructures due to the much lower mobility of this disordered material. The differences in the lateral conductivity for the triclinic and orthorhombic rubrene originate from the highly anisotropic transport in the triclinic crystals, preferring vertical rather than lateral transport. Furthermore, it should be noted that in comparison to the impedance study, we extended the range of dopant concentration to 40 mol % for the conductivity analysis since higher conductivity values are preferred for efficient thermoelectric devices. These dopant concentrations though are too high for the impedance analysis as they would result in very thin depletion zones that cannot be resolved anymore. However, we assume that the values for the doping efficiency in modulation-doped devices do not vary significantly with the dopant concentration.

The Seebeck coefficient is measured by applying a lateral temperature gradient across the sample using two separated copper blocks with independent heating unit and measuring the thermo-voltage generated. A typical measurement routine is shown in fig. S4, and the thermo-voltage measured by taking the voltage difference by applying temperature gradient in opposite direction that is useful to exclude any nonthermoelectric offset voltage and the Seebeck coefficient can be taken as Δ*V*/Δ*T* for the mean temperature. The Seebeck coefficient for the modulation-doped orthorhombic rubrene crystals reduces with increasing doping concentration, which is consistent with increasing the free charge carrier density in rubrene that shifts the chemical potential toward the transport edge (Fig. 2B) (*30*, *31*). The thermoelectric power factor (*S*^2^σ) is a key figure of merit for the performance of thermoelectric materials (*32*, *33*). For modulation-doped orthorhombic rubrene crystals, the thermoelectric power factor generally increases with the doping concentration, reaching a peak value of 6.9 ± 1.9 μW/K^2^/m^1^ at around 30 mol % of NDP-9 doping at room temperature (Fig. 2C).

Although the doping efficiency of the modulation-doped rubrene crystals with different phase is similar, the Seebeck coefficient for the triclinic crystal phase is more than twice higher compared to the orthorhombic crystal phase at the same dopant concentration. To rule out the possibility that the difference comes from the degree of disorder of the two crystal phases, we have measured the Urbach energy (*U*_e_) of the two crystal phases using photothermal deflection spectroscopy. The values of Urbach energy are similar (*U*_e_ = 74 ± 3 meV for orthorhombic rubrene thin-film crystals and *U*_e_ = 82 ± 4 meV for triclinic rubrene thin-film crystals), which is expected as both crystal phases form large domains of highly ordered crystals (Fig. 2D) (*26*, *27*). The difference between the Urbach energy of the rubrene thin-film crystal and bulk single crystal [*U*_e_ ~ 40 meV (*34*)] is probably due to the additional disorder associated with the polycrystalline nature of the thin-film crystals. Therefore, the difference in Seebeck coefficient observed is probably not due to energetic disorder but related to the local energy landscape that charge carriers experience in triclinic rubrene crystals (*35*, *36*).

We further investigate the degree of disorder in rubrene crystals associated with modulation doping using two methods. We first look at the activation energy of electrical conductivity that could provide information on the degree of disorder in the host-dopant system. In the modulation-doped crystals, the activation energy of the electrical conductivity remains relatively constant (fig. S5), indicating minimal structural and/or electrostatic disorder at the rubrene crystal/doped BPAPF interface (*6*, *21*, *31*). Moreover, we characterized top-gated transistors with top contacts based on modulation-doped orthorhombic rubrene crystals to understand the charge transport in rubrene crystal upon doping. We have carefully designed the dielectric materials and their thicknesses to maximize the dielectric capacitance to allow the possibility to modulate the charge carrier density in rubrene crystals with very high carrier density. The thin CYTOP polymer layer was introduced between the rubrene and AlO*_x_* layer to avoid damage to the rubrene surface by exposure to the atomic layer deposition precursors (*37*). Figure S2 shows the transfer curves of the modulation-doped rubrene thin-film crystal transistor with a poor on/off ratio that is consistent with the presence of a high free charge carrier density. However, the linearity of the transfer curve allows the extraction of field-effect carrier mobility from the linear regime of the transfer curve. The field-effect mobility for 40 mol % modulation-doped rubrene crystal was estimated to be around 0.1 cm^2^ V^−1^ s^−1^. Although not optimum, it is comparable to other large-area polycrystalline orthorhombic crystal devices prepared on poly(methyl methacrylate) dielectric films (*26*). We would like to point out that the top-gated transistor with top contact configuration is generally not ideal since this limits the charge injection from the source and drain electrodes. In addition, the doped BPAPF layer also further limits efficient charge injection from the source and drain electrodes due to its deep HOMO level and low charge carrier mobility. Therefore, our results indicate that electrostatic disorder is unlikely to dominate the charge carrier transport in rubrene crystals doped by modulation doping.

### Theoretical simulation of thermoelectric transport

**Fig. 3.**
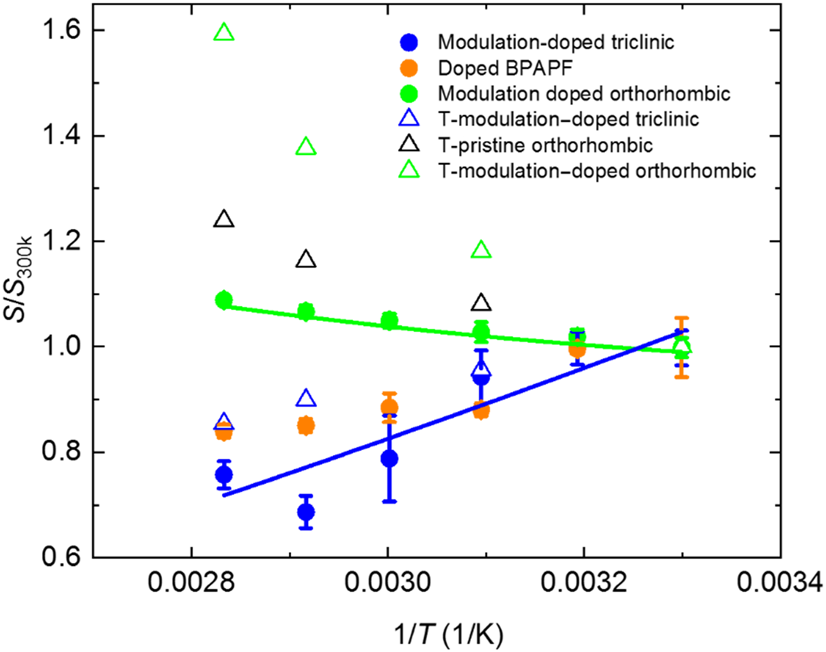
Temperature-dependent Seebeck coefficient. Theoretically simulated Seebeck coefficient as a function of inverse temperature of undoped orthorhombic (black open triangles), modulation-doped orthorhombic (green open triangles), and modulation-doped triclinic (blue open triangles) rubrene samples. The simulation was taken at the charge neutrality point (*n*_h_ = 16% of holes in the system). The solid circles are experimental Seebeck coefficient as a function of temperature for modulation-doped triclinic rubrene (40 mol % NDP-9–doped BPAPF, blue), doped BPAPF (40 mol % NDP-9 doping, orange), and modulation-doped orthorhombic platelet rubrene (40 mol % NDP-9–doped BPAPF, green) samples. The blue and green solid lines are guides for the eye.

The temperature-dependent Seebeck coefficient in doped organic semiconductors is governed by both the thermal activation of the dopant-host charge-transfer complexes (*6*, *21*) and the charge transport physics of the host molecules and can therefore provide deep insights into the carrier transport mechanisms in organic semiconductors (*30*, *35*). Figure 3 shows a compilation of the theoretical and experimental temperature-dependent Seebeck coefficients of modulation-doped rubrene crystals (see Materials and Methods for details on the simulations). The modulation-doped triclinic rubrene crystals show a decrease in the Seebeck coefficient with increasing temperature. This trend is commonly observed in bulk-doped organic semiconductors since temperature increase leads to thermal activation of integer charge-transfer complexes, which allows an increased probability for free charge carrier release into the host semiconductor, and hence, the transport level moves closer to the chemical potential (for comparison, data for bulk-doped BPAPF are shown as orange solid circles). On the other hand, the modulation-doped orthorhombic rubrene crystal shows an unexpected increase in the Seebeck coefficient with increasing temperature. To understand the origin of this trend, we have carried out simulations on the carrier concentration–dependent Seebeck coefficient at different temperatures (see Materials and Methods for details).

**Fig. 4.**
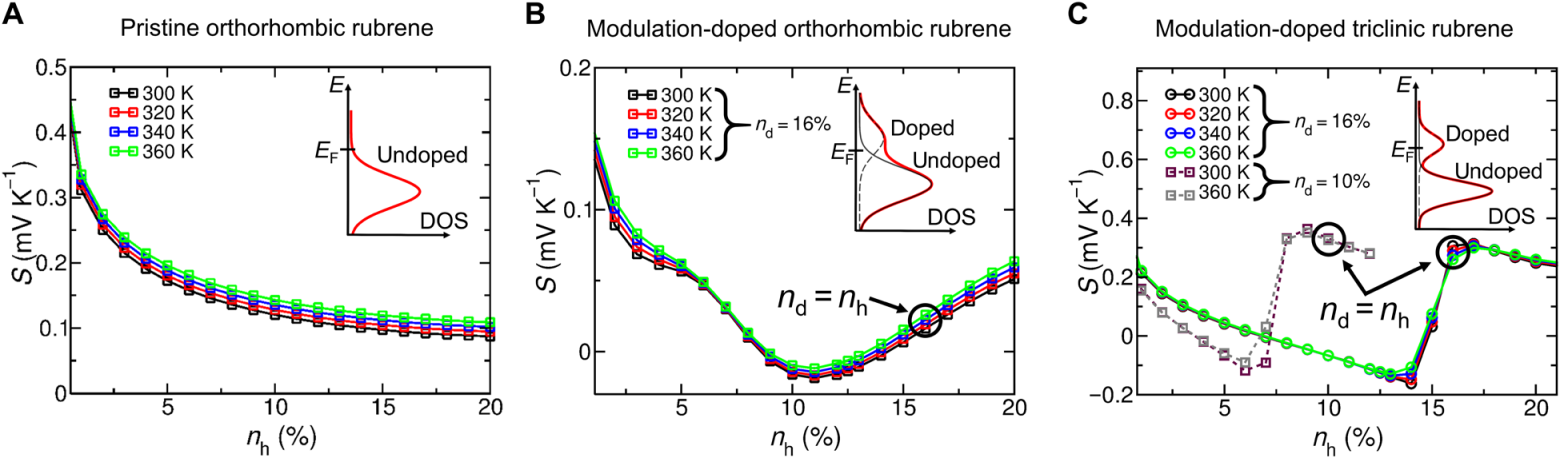
Theoretical simulation of thermoelectric transport in crystalline rubrene thin films. (**A**) Seebeck coefficient as a function hole density *n*_h_ for pristine orthorhombic rubrene crystals (without counter ions). (**B**) Seebeck coefficient as a function of hole density *n*_h_ for modulation-doped orthorhombic rubrene crystals with a doping concentration of *n*_d_= 16% and (**C**) modulation-doped triclinic rubrene crystals with lower electronic coupling for doping concentrations of *n*_d_= 10% and *n*_d_= 16%. The charge neutrality point is indicated by the large black circles. The insets show the DOS around the Fermi energy (*E*_F_).

Figure 4 (A to C) shows the simulated Seebeck coefficient as a function of the carrier concentration *n*_h_ at various temperatures for pristine orthorhombic rubrene crystals, modulation-doped orthorhombic rubrene crystals, and modulation-doped triclinic rubrene crystals, respectively.

For the pristine crystals (Fig. 4A), the Seebeck coefficient decreases with increasing carrier concentration, which is due to the shift in the chemical potential toward the transport level with increasing free carrier density. A negative Seebeck coefficient is only expected above half filling of the HOMO-derived states of the pristine rubrene crystals but not in the plotted density range.

For the modulation-doped systems, we first select a doping concentration of *n*_d_= 16% for the orthorhombic and the triclinic systems and study *S*(*n*_h_) in Fig. 4 (B and C). At small carrier densities, the Seebeck coefficient also reduces with increasing carrier density, but, in contrast to the pristine case, at higher carrier density, the Seebeck coefficient becomes negative and the slope with *n*_h_ is reversed.

This behavior can be attributed to the influence of the doped BPAPF at the interface. The behavior of the Seebeck coefficient of the modulation-doped systems is mainly determined by the density of states (DOS) of the HOMO, which is explained as follows: Because of the interaction of the holes in rubrene with the counterions in the doping layer, the HOMO levels at the interface are shifted toward lower binding energies (see Materials and Methods for details on modeling). Owing to the weak dielectric screening, the energy difference between the doping-induced interface states and the unaffected bulk states may result in an energy gap Δ or a minimum in the energy dependence of the DOS of the modulation-doped system as seen in the insets of Fig. 4 (B and C). This minimum appears in the energy region between doping-induced interface states (a fraction of all hole states) and bulk states. As a result, the observed inversion of the Seebeck coefficient appears when the Fermi level lies between these doping-induced and bulk hole states. We find negative values for the Seebeck coefficients when the Fermi level surpasses the doping-induced states when the impact of the bulk states is not yet relevant (see Fig. 4, B and C, at *n*_h_≈ 9 to 12%). The predicted sign inversion of the Seebeck coefficient at very high carrier density due to the influence of the dopant acceptor levels on the overall blend film energetics has recently been verified (*38*). The DOS in the discussed energy region is different for the modulation-doped orthorhombic and the modulation-doped triclinic system, leading to a different *S*(*n*_h_) behavior. This is analyzed in more detail further below.

We first want to relate the theoretical results to the measurements in Fig. 2B. Toward this end, we additionally simulate the Seebeck coefficients for the modulation-doped triclinic system for a lower doping concentration of *n*_d_= 10% (see Fig. 4C), which shows the same qualitative behavior as observed at *n*_d_= 16%, including the inversion of the Seebeck coefficient. We can compare both simulations at their respective charge neutrality point, i.e., at *n*_d_ = *n*_h_ (see large black circles in Fig. 4C), which is more directly comparable to the measurements. We observe that the Seebeck coefficient at charge neutrality changes only slightly when the doping concentration is decreased. This observation is in full consistence with the experimental results for the doping concentration–dependent Seebeck coefficient of the modulation-doped system shown in Fig. 2B.

Next, we want to explain in more detail the experimental results for the temperature dependence of the Seebeck coefficient of the modulation-doped triclinic system in Fig. 3, and we have analyzed the behavior close to charge neutrality in more detail. The inversion of the temperature dependence at the charge neutrality point (large black circles in Fig. 4C) for the triclinic system is rationalized with the energy gap between doping-induced and bulk hole states and depends on the electronic bandwidth associated with both sets of states. If the energy gap Δ exceeds the electronic bandwidths [for a Gaussian DOS, the bandwidth is defined as the standard deviation (SD) σ] of the doping-induced and the bulk hole states (see the inset of Fig. 4C for an illustration), then the Seebeck coefficient grows approximately with exp((Δ − σ)/*k*_B_*T*). Thus, for a broad and deep minimum in the DOS, the Seebeck coefficient increases with decreasing temperature, i.e., its ordinary temperature dependence is inverted, as seen for the modulation-doped triclinic system in Figs. 3 and 4C near *n*_d_ = *n*_h_ ≈ 16% (and similarly at *n*_d_ = *n*_h_≈10%). In contrast, this effect is suppressed for the modulation-doped orthorhombic system, due to the enhanced electronic coupling in the latter case that leads to a broader electronic bandwidth and hence smaller width (or absence) of the minimum in the DOS (see the inset of Fig. 4B for illustration). In summary, the more pronounced energy minimum in the DOS of the triclinic system causes a more abrupt change in the Seebeck coefficient near the charge neutrality point as seen in Fig. 4C and leads to the different temperature dependencies of the Seebeck coefficient between the modulation-doped orthorhombic and triclinic rubrene crystals. We further note that the much larger electronic coupling in orthorhombic crystals as compared to triclinic rubrene crystals caused a lower lateral carrier mobility of the latter in consistence with the experimental mobilities in Fig. 2.

Although the absolute values of the Seebeck coefficient do not quantitatively match between theory and experiment, the trends regarding temperature dependence and the differences between the samples are qualitatively consistent. The quantitative difference is possibly associated with the thermal activation of the carrier concentration due to the more efficient charge release from the charge-transfer complexes at elevated temperatures that has not been taken into account in the theoretical model. The increasing Seebeck coefficient with increasing temperature in modulation-doped orthorhombic rubrene crystal allows the thermoelectric power factor to reach 20.6 ± 3.8 μW m^−1^ K^−2^ at 80°C, which is comparable to other best-performing organic polymer thermoelectric materials (*32*, *33*).

To achieve large (positive or negative) Seebeck coefficients near the charge neutrality point, it is beneficial to tune the energetics of the heterostructure such that the doping-induced levels have a narrow energy distribution. A moderate interface coupling would then ensure the injection of charges into the undoped layer without affecting the Seebeck coefficient too much. For this case, small changes in the temperature and in the carrier concentration might cause significant changes in *S* if the Fermi level is located near the minimum of the DOS of the modulation-doped system.

In summary, we investigated the charge and thermoelectric transport in modulation-doped large-area rubrene thin-film crystals with different crystal phases. We demonstrated superior doping efficiency over 20% in modulation-doped rubrene thin-film crystals, where both high degree of molecular order and doping efficiency are preserved. High thermoelectric power factors exceeding 20 μW m^−1^ K^−2^ at 80°C can be achieved with modulation-doped orthorhombic rubrene crystals. This achievement, underpinned by the theoretical insights on the energy landscape of the heterostructures and its influence on qualitative trends of the Seebeck coefficient, opens up prospective for using modulation doping together with high-mobility crystalline organic semiconductors as a strategy for achieving high-performance organic thermoelectrics.

## MATERIALS AND METHODS

### Sample preparation

One-millimeter-thick glass wafers were cleaned in acetone and isoproponal for 10 min under ultrasonication followed by oxygen plasma cleaning at 100 W for 5 min. For triclinic rubrene crystalline thin film, 50 nm of rubrene was thermally evaporated onto the substrate under ultrahigh vaccum at a base pressure better than 1 × 10^−7^ mbar followed by thermally annealing on a hot plate under nitrogen atmosphere with water and an oxygen level below 0.1 parts per million at 130°C for 15 min. For orthorhombic rubrene crystalline thin film, a bilayer of 1,1-Bis[(di-4-tolylamino)phenyl]cyclohexane (TAPC) (5 nm) and rubrene (40 nm) was deposited on the substrate and annealed on the hot plate at 160°C for 4 min. The thermal annealing conditions allow the crystallite domains with hundreds of micrometer in size to fully cover the surface of the substrate. The wafers were then moved back to the deposition chamber to evaporate 30 nm of NDP-9–doped BPAPF on top of rubrene followed by gold electrode deposition (the gold electrodes has a channel length of 5 mm and a width of 20 mm). For the transistor fabrication, 5 nm of the NDP-9–doped BPABF was deposited on top of rubrene crystalline thin film followed by Au electrode deposition (the channel length is 100 μm and the width is 1 mm). The doped layer was made as thin as possible to ensure successful depletion of the doped layer and charge accumulation in the rubrene layer. CYTOP (65 nm) was spin-coated on top of the device at a speed of 1000 rpm for 1 min and subsequently annealed at 90°C for 20 min. Fifty nanometers of aluminum oxide was then coated on the device using atomic layer deposition at 70°C (the capacitance of the dielectric layer is calculated to be 23 nF/cm^2^). The Schottky diodes used for Mott-Schottky analysis have an active area of 200 μm by 200 μm.

### Measurements

The Seebeck measurement was performed by placing the sample on separated copper blocks with embedded thermal element and contacting thermal couples at thermally equivalent position to the electrical contacts. The Seebeck measurements were carried out under vacuum at a pressure of around 1 × 10^−6^ mbar, and the thermal voltage was probed by Keithley 236 measurement unit. The conductivity of the modulation-doped devices was averaged over three devices, assuming that the conductivity mainly occurs at the interface [thickness taken as 1.5 nm (a monolayer of rubrene molecules)]. This hypothesis is validated by changing the thickness of rubrene and/or NDP-9–doped BPAPF, which yields negligible resistance change. The Seebeck coefficients were averaged over at least five measurements. The transistor measurements were taken inside a nitrogen glovebox using two Keithley 236 measurement units at room temperature. The transistor performance is averaged over five devices. The capacitance measurements are measured with a HP 4284A measurement unit. The Schottky diodes were encapsulated under a nitrogen environment. For the doping efficiency calculation, the molecular density of BPABF is estimated to be 7 × 10^20^ cm^−3^ using first-principle calculation. For the photothermal deflection spectroscopy (PDS) measurements, the samples were placed into a sample holder filled with Fluorinert FC-770 (3M). The PDS setup uses a 150-W Xenon short-arc lamp (Ushio) that provides light for a monochromator (Oriel Cornerstone, 16 nm full width at half maximum) to achieve a chopped, tunable, and monochromatic pump beam. The heat caused through absorption of the pump light in the film changes the refractive index of the Fluorinert. This change is detected by deflecting a diode laser (635 nm; Thorlabs) whose displacement is measured by a position sensitive detector (Thorlabs PDP90A). The magnitude of the deflection is determined by a lock-in amplifier (Amatec SR 7230) and directly correlated to the absorption of the film.

### Theoretical modeling

To study the thermoelectric transport in pristine and modulation-doped crystalline rubrene thin films, we use a theoretical model involving vibronic material parameters for electronic coupling and thermal disorder due to intramolecular electron-phonon interaction. The underlying model Hamiltonian

$$\hat{H}=\sum_{\mathrm{MN}}(E_{\mathrm{MM}}^{\text{HOMO}}+V_{\mathrm{MM}}^{\text{th}}){\hat{c}}_{M}^{†}{\hat{c}}_{M}+\varepsilon_{\mathrm{MN}}{\hat{c}}_{M}^{†}{\hat{c}}_{N}$$ (3)

describes the energy levels of HOMO of the doping layer and the rubrene layer via $E_{\mathrm{MM}}^{\text{HOMO}}$, the vibration-induced thermal disorder via $V_{\mathrm{MM}}^{\text{th}}$, and the HOMO dispersion via ε*_MN_*. The vibronic material parameters for the orthorhombic rubrene crystal phase and the BPAPF single molecule are used from the literature (*39*, *40*).

The electronic states in rubrene at the interface are affected by the dopants in the adjacent doped layer due to hole-counterion attraction. This interaction depends strongly on the distance to the interface (*41*, *42*). Since the spacing of the rubrene molecules along the phenyl side chains perpendicular to the doping layer is large (lattice constant of around 27 Å for orthorhombic rubrene) and, thus, the hole binding energy is small or even negligible in the bulk, we consider the binding energy of the holes and the counterions to be relevant only inside the interface layer. Therefore, we distinguish the energy position between whether the rubrene molecules are located in the interface layer or in the bulk (step profile) and shift the HOMO energies at the interface layer by a constant value. We set the energy difference between the HOMO energies of the rubrene molecules in the bulk and inside the interface layer to 0.4 eV, which is typical for molecular systems (*41*, *42*).

For both the modulation-doped orthorhombic and triclinic system, we assumed a doping layer with a molar ratio of 0.16 (and additionally 0.10 for triclinic rubrene only) of the total system size of around 5 × 10^7^ HOMO orbitals. The layer thickness of the model system amounts to 8.1 nm (5.4 nm, triclinic only) for the doped layer and 45.9 nm (48.6 nm, triclinic only) for rubrene layer with a lateral extent of about 1500 nm by 800 nm. In our model calculations, we have not considered any effects from grain boundaries.

The Seebeck coefficient for the HOMO is calculated from the dc conductivity as

$$S(E_{F})=\frac{k_{B}}{e}\int \mathrm{dE}\frac{\left(E_{F}-E\right)}{k_{B}T}\frac{\sigma (E)}{\sigma }$$ (4)

with the electrical conductivity

$$\sigma =\int \mathrm{dE}\sigma (E)=\int \mathrm{dEf}(E-E_{F})(1-f(E-E_{F}))D(E)\mu (E)$$ (5)

that is proportional to the DOS *D*(*E*) and the mobility μ(*E*) of a single HOMO hole (*30*, *43*). The Fermi function *f*(*E* − *E*_F_) refers to the occupation of the HOMO in the doped system. The dc conductivity is calculated using a real-space implementation of the Kubo formula (*44*, *45*), where electronic correlations are neglected and the carrier mobility satisfies an Einstein relation according to

$$\mu (E)=e/k_{B}T\underset{t\to \infty }{\text{lim}}\Delta x^{2}(E,t)/2t$$ (6)

with the mean squared deviation Δ*x*^2^(*E*, *t*) of a single carrier divided by the correlation time *t*. The correlation time *t* ranges up to 1.5 ps in the performed simulations. Besides the calculations of thermoelectric transport of doped rubrene crystals, we also simulated the thermoelectric transport of the undoped orthorhombic crystal phase.
