# Supplementary material for: Highly efficient modulation doping: A path toward superior organic thermoelectric devices
Source: Sci Adv. 2022 Mar 30;8(13):eabl9264. doi: 10.1126/sciadv.abl9264 (PMC8967228; doi:10.1126/sciadv.abl9264)
Supplement: Supplementary file 1 — Figs. S1 to S7 [file sciadv.abl9264_sm.pdf]

Science Advances
AAAS

Supplementary Materials for

Highly efficient modulation doping: A path toward superior organic thermoelectric devices

Shu-Jen Wang*, Michel Panhans, Ilia Lashkov, Hans Kleemann, Federico Caglieris, David Becker-Koch, Jörn Vahland, Erjuan Guo, Shiyu Huang, Yulia Krupskaya, Yana Vaynzof, Bernd Büchner, Frank Ortmann*, Karl Leo*

*Corresponding author. Email: shu-jen.wang@tu-dresden.de (S.-J.W.); frank.ortmann@tum.de (F.O.); karl.leo@tu-dresden.de (K.L.)

Published 30 March 2022, *Sci. Adv.* **8**, eabl9264 (2022)
DOI: 10.1126/sciadv.abl9264

This PDF file includes:

Figs. S1 to S7

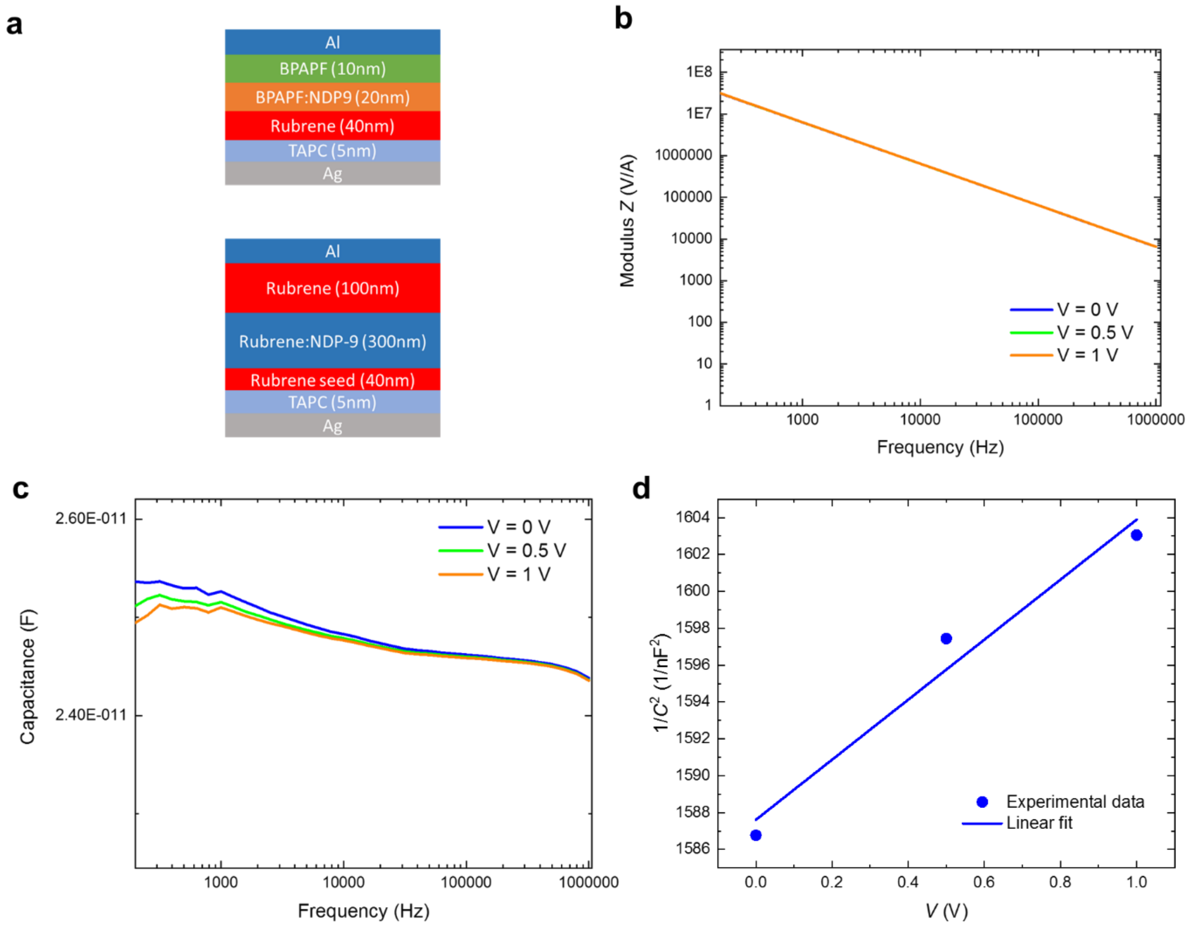

Fig. S1. Mott-Schottky analysis of modulation doped films. (**a**) Schematic illustration of the Schottky diode layer stacks for Mott-Schottky analysis. Modulus (**b**) and capacitance (**c**) as a function of frequency for a modulation doped triclinic rubrene crystal based Schottky diode under different external applied voltages. The modulus versus frequency shows the ideal $1/f$ relation as a standard RC element. The external voltage is applied on the Al electrode to drive the diode in reverse direction. (**d**) Mott-Schottky fit for modulation doped triclinic rubrene crystals.

Description of Impedance Analysis:

The Schottky and heterojunction diodes are characterized by impedance spectroscopy in the frequency range between 100Hz and 1MHz. In order to suppress undesired leakage currents impeding the impedance analysis, thin intrinsic buffer layers of rubrene and BPAPF have been added to the stack. In consequence, the impedance response of the junctions is not only governed by the response of the charge depletion zone but also contains contributions from the intrinsic layers. As the width of the charge depletion zones is usually small (<20nm), the total capacitance of the junction is governed by the capacitance of the intrinsic layers. In order to account for this situation, we describe the junctions by an equivalent circuit containing two RC-parallel elements. The first element describes the voltage dependent depletion capacitance, while the second element accounts for the intrinsic layer capacitance. Due to the choice of the thickness of the intrinsic layers, the capacitance of the intrinsic layer appears in the high-frequency part of the impedance spectrum (>5kHz) showing no voltage dependence. The low-frequency plateau (<5kHz) describes the charge depletion zone due to the doping and the voltage dependence of the capacitance can be used for the Mott-Schottky analysis (taken at 5kHz).

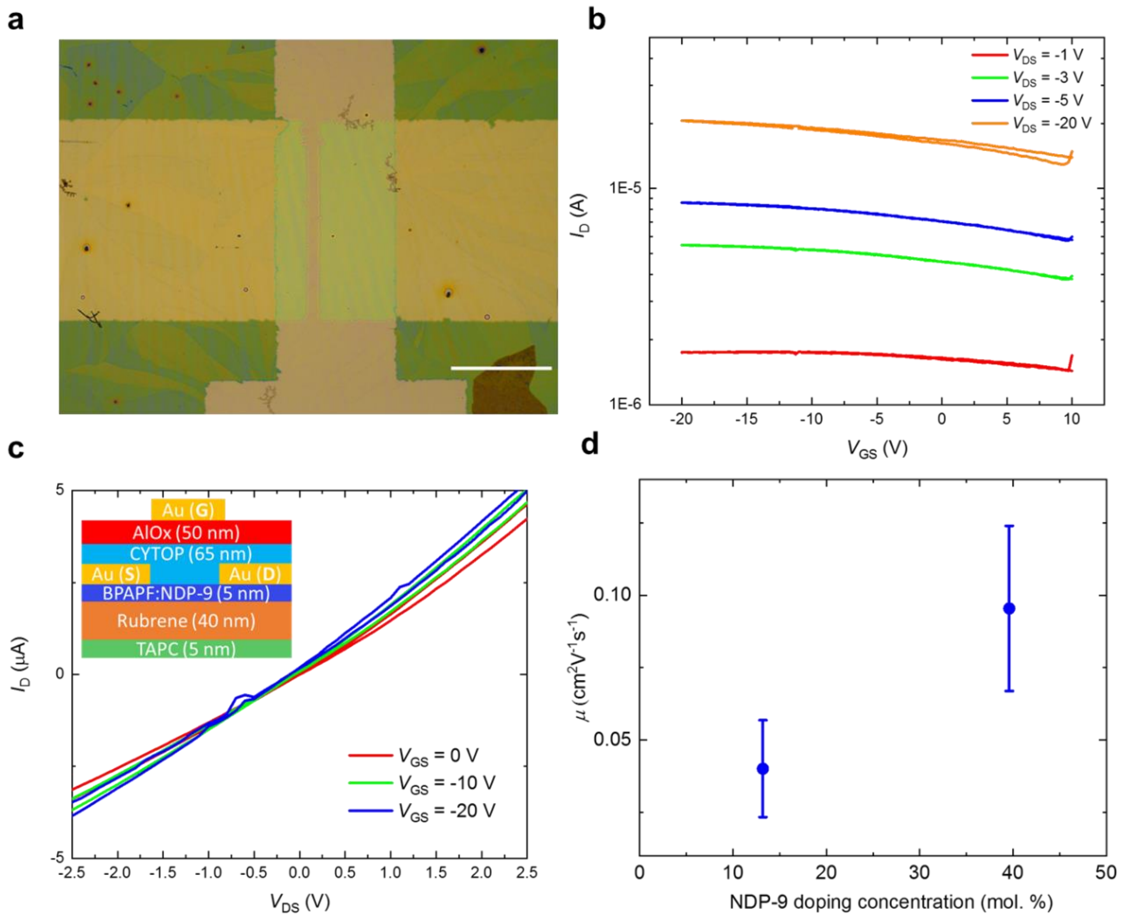

Fig. S2. Transistor characterization of the modulation doped rubrene devices. (**a**) Optical microscope image of typical modulation doped rubrene transistor device (top gate and top contacts). The channel length is 100 µm and width 1 mm. The scale bar corresponds to a size of 500 µm. Transfer (**b**) and output (**c**) characteristics of modulation doped orthorhombic rubrene thin-film crystal transistor measured at room temperature. The inset illustrates the layer stack of the transistor. (**d**) The carrier mobility estimated from the linear regime of the transistors under different BPAPF:NDP-9 doping concentration.

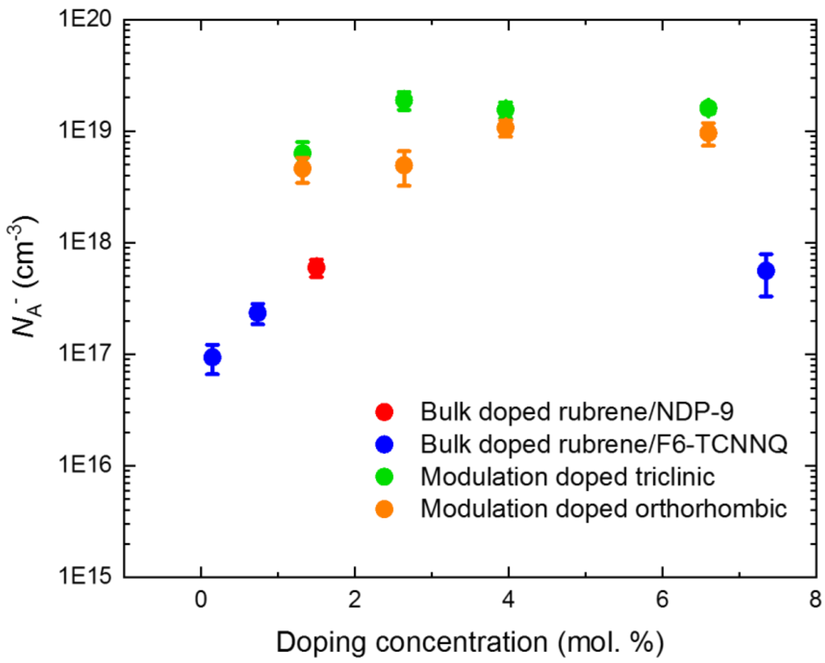

Fig. S3. Ionized dopant density with doping concentration. Ionized dopant, N_A^- as a function of dopant molar concentration determined from Mott-Schottky analysis. The green and orange circles denote modulation doped triclinic and orthorhombic rubrene thin-film crystals with BPAPF:NDP-9 respectively. The red circle represents bulk doped orthorhombic rubrene thin-film crystals with NDP-9. The bulk doped triclinic rubrene/F6-TCNNQ data (blue circles) were taken from [21] for comparison.

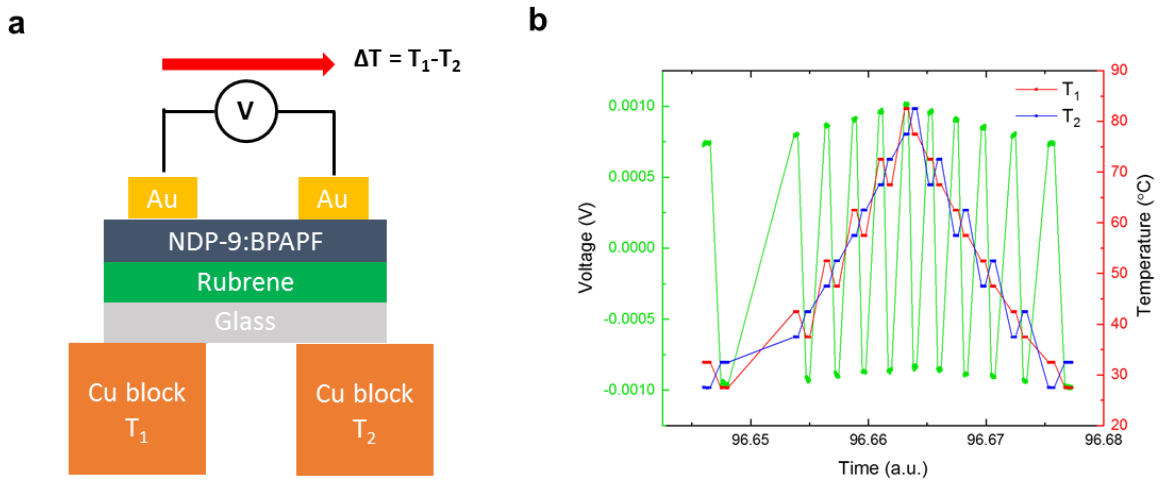

Fig. S4. Seebeck measurement routine. (**a**) Schematic illustration of the Seebeck measurement geometry. (**b**) Typical time dependent Seebeck measurement at different temperatures. T_1 and T_2 denote the temperatures of independent separated copper blocks.

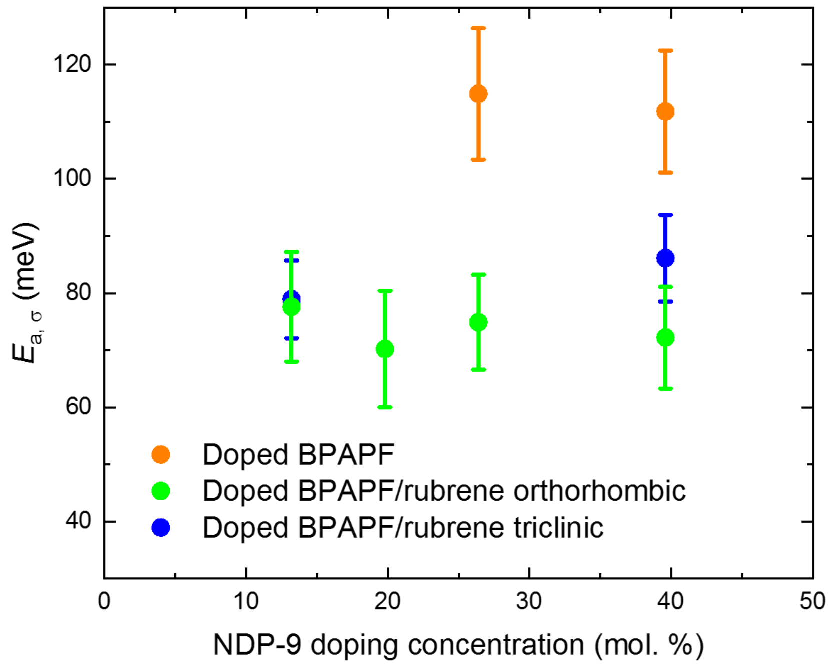

Fig. S5. Activation energy of electrical conductivity with doping concentration. Activation energy of electrical conductivity dependence on the doping concentration. The orange solid circles is for bulk doped BPAPF thin films while the green circles is for modulation doped orthorhombic rubrene crystals and the blue circles is modulation doped triclinic rubrene crystals.

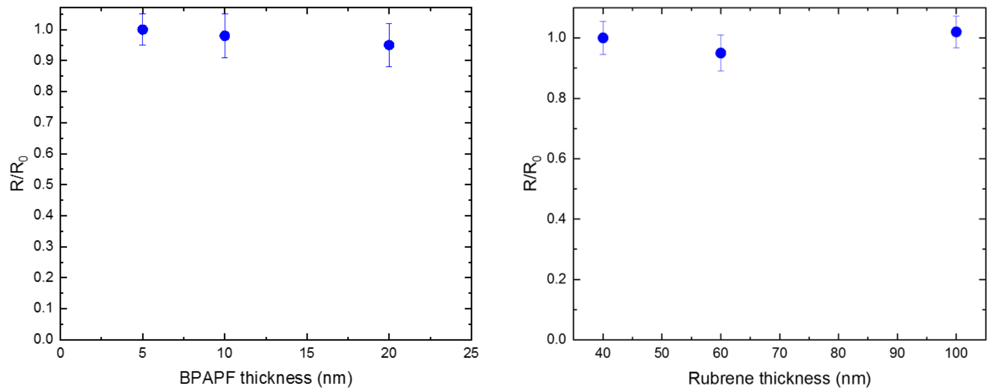

Fig. S6. Thickness dependence of the modulation doped rubrene device resistance. Thickness-dependent normalized device resistance (to device with 5 nm BPAPF (doped with 10 wt.% NDP-9) and 40 nm rubrene). The channel length is 100 um and channel width is 1 mm. The error bars denote the standard deviation from averaging several devices.

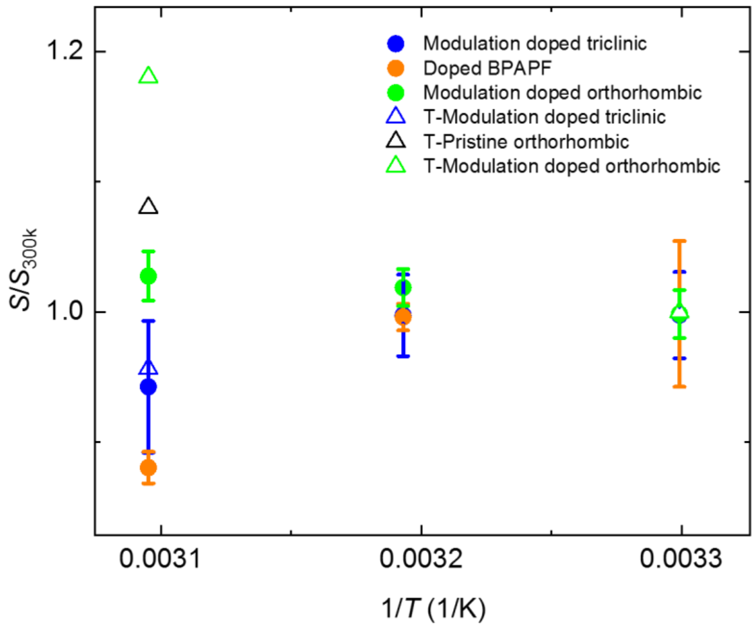

Fig. S7. Zoomed in illustration of the temperature dependent Seebeck coefficient. Zoomed in temperature-dependent theoretical and experimental Seebeck coefficient as Fig. 3 to make the data points more distinguishable at low temperature regime.
